# Switching from Intravitreal Ranibizumab to Bevacizumab for Age-Related Macular Degeneration

**DOI:** 10.5402/2011/916789

**Published:** 2011-12-21

**Authors:** Kisaburo Yamada, Kenichi Kimoto, Hirofumi Kono, Toshiaki Kubota

**Affiliations:** Department of Ophthalmology, Faculty of Medicine, Oita University, Hasama-machi, Yufu-shi, Oita 879-5593, Japan

## Abstract

*Purpose*. To report our experiences in patients with age-related macular degeneration (AMD) treated initially with intravitreal ranibizumab and then switched to bevacizumab. *Methods*. We retrospectively reviewed the records of 7 patients (7 eyes) who were treated with monthly injections of intravitreal ranibizumab and then switched to injections of bevacizumab (every 6 weeks) for six months. The best-corrected visual acuity measurements (BCVA) and optical coherence tomography (OCT) were performed at the baseline examination and then at each visit. The Wilcoxon signed-rank test was used for the statistical analysis. *Results*. Following three monthly ranibizumab treatments, there was no significant difference in the BCVA, while the foveal retinal thickness (FRT) significantly decreased (*P* < 0.01). Switching from ranibizumab to bevacizumab resulted in maintenance (57.2%) of the BCVA and a further decrease in the FRT (*P* < 0.01) after 6 months. *Conclusions*. Switching to intravitreal bevacizumab may be effective in patients who wish to discontinue intravitreal ranibizumab treatment due to the high cost.

## 1. Introduction

Age-related macular degeneration (AMD) is the leading cause of severe visual loss in patients over 60 years of age [1]. The development of vascular endothelial growth factor (VEGF) antagonists was a landmark in the treatment for the exudative form of this disease. Currently, the most commonly used VEGF antagonists are ranibizumab (Lucentis; Genentech, San Francisco, California, USA) and bevacizumab (Avastin; Genentech). Both molecules are derived from the same murine monoclonal antibody against VEGF. Ranibizumab, the corresponding Fab fragment of the full-length anti-VEGF antibody, was specifically designed and approved for intravitreal treatment of exudative AMD [2]. Controlled studies with ranibizumab have since dramatically increased the expectations for visual outcome [3–5]. Bevacizumab, a humanized monoclonal full-length antibody to VEGF, is approved for the systemic intravenous treatment of metastatic colorectal cancers [6] but is not currently approved for injection into the eye. The first report of intravitreal bevacizumab administration for neovascular AMD was published in 2005 [7]. This “off-label” use of the drug has been reported to be effective for exudative AMD [8–12]. Although the current treatment for exudative AMD is ranibizumab, there are some cases where the intravitreal ranibizumab treatment must be discontinued due to its high cost [13, 14]. 

 We herein report our experiences with patients treated initially with intravitreal ranibizumab and then switched to bevacizumab because of the expensive cost of ranibizumab for a followup period of at least 6 months.

## 2. Materials and Methods

This was a retrospective study of patients with exudative AMD of all subtypes, and with signs of active CNV with decreased visual acuity and leakage in fluorescein angiography (FA). A total of 128 patients (129 eyes) were treated initially with intravitreal ranibizumab at 0.5 mg/0.05 mL for three months (three monthly injections), and then 11 patients (11 eyes) switched to bevacizumab at 1.25 mg/0.05 mL for six weekly injections. The mean age of the 128 patients was 70.6 ± 7.1 years (range 61–80). Four of 11 patients switched because of the insufficient efficacy of ranibizumab, and seven of the 11 patients switched because of the high cost of ranibizumab. The patients, who had undergone photodynamic therapy with Visudyne before starting the anti-VEGF treatment, were also included in the study. 

Patients were monitored for their decimal best-corrected visual acuity at 5 m (BCVA). Optical coherence tomography (OCT) was performed at each visit. The foveal retinal thickness (FRT), measured in micrometers, was determined with the use of a OCT-1000 instrument (version 3.01, Mark II; Topcon Corporation, Tokyo, Japan) and included descriptions of intraretinal cysts, subretinal fluid, and retinal pigment epithelial detachment. 

 In all cases, when changing from ranibizumab to bevacizumab, the first bevacizumab injection was performed one month after the last ranibizumab injection in order to avoid a time interval delay in which the eye was not “covered” by any anti-VEGF treatment. All injections were performed under topical anesthesia and standard sterile conditions. Topical antibiotics were administered for three days. Re-treatment was performed if visual acuity deteriorated with new intraretinal or subretinal fluid in the OCT. All patients signed an informed consent form after receiving a detailed explanation of the procedure. The intravitreal injection of bevacizumab was approved by the Oita University Hospital Ethics Committee. The decimal visual acuity was converted to a logarithm of the minimal angle of resolution (log⁡⁡MAR) for statistical reasons. The Wilcoxon signed-rank test was used for the statistical analysis. A *P* value < 0.05 was considered to be statistically significant.

## 3. Results

Seven patients (7 eyes) were switched to bevacizumab from ranibizumab because of the expensive cost of ranibizumab. The mean age of the patients was 70.7 ± 7.1 years (range 62–79). Of the 7 patients, 2 were male (28.6%) and 5 were female (71.4%). All patients completed at least 6 months of followup, and the mean followup period was 10.6 ± 1.3 months (range 9–13 months) (Table 1).

 Following the intravitreal ranibizumab, the BCVA increased from 0.40  log⁡⁡MAR before the first treatment to 0.20  log⁡⁡MAR (*P* > 0.05) after the three monthly injections (4 months after the first treatment). The mean FRT as measured by OCT decreased from 406.6 *μ*m (±148.1 *μ*m) before treatment to 285.6 *μ*m (±129.4 *μ*m) (*P* < 0.01) after three monthly injections. Although continuous treatments were necessary, seven patients complained of difficulty to continue the intravitreal ranibizumab treatment because of its high cost. Therefore, we changed the treatment from ranibizumab to bevacizumab for these patients.

**Figure 1:**
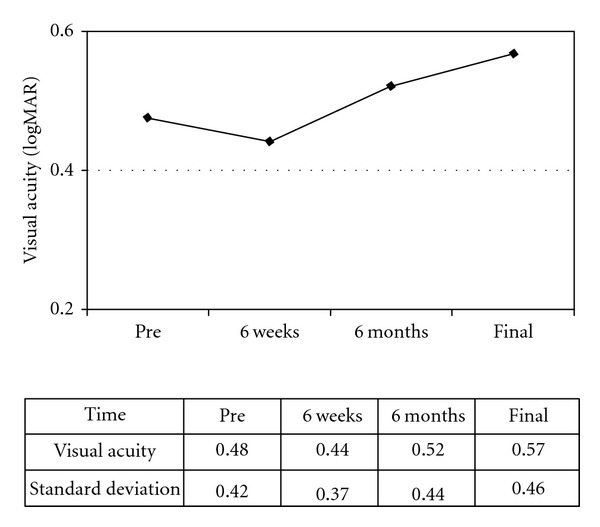
The mean log⁡⁡MAR of the best-corrected visual acuity (BCVA) at each of the visits. There were no statistical significant differences between any of the points and the preswitching value.

**Figure 2:**
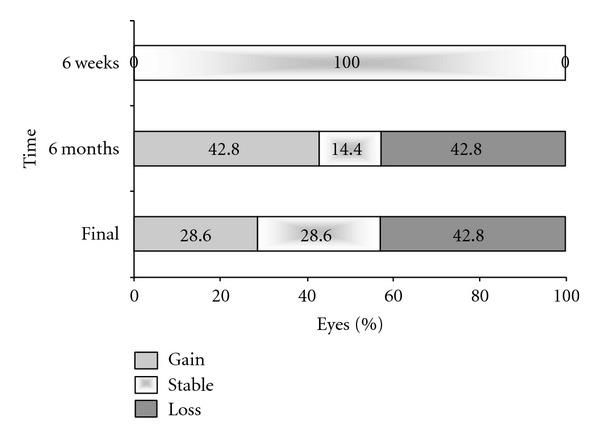
The changes in the mean log⁡⁡MAR BCVA at the followup visits.

**Figure 3:**
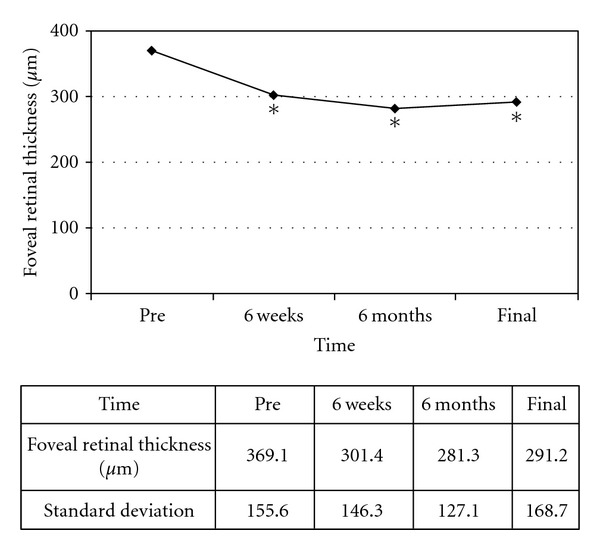
The mean foveal retinal thickness (FRT) at the followup visits and prior to switching treatment. A significant difference was noted at six weeks, six months, and at the final visit compared with the value before the switch (*The Wilcoxon signed-rank test: *P* < 0.01).

 The BCVA changed from 0.48  log⁡⁡MAR before the first intravitreal bevacizumab to 0.44  log⁡⁡MAR (*P* > 0.10) after six weeks and was 0.52  log⁡⁡MAR (*P* > 0.05) after six months and 0.57  log⁡⁡MAR (*P* > 0.10) at the last followup—termed the ‘‘final” followup in the analysis. There were no statistically significant differences between each point and that prior to the switch (Figure 1). To evaluate the change in the BCVA, we defined stable visual acuity as a gain or loss of <0.3  log⁡⁡MAR units. A stable BCVA or a gain of BCVA was achieved in 100% (6 weeks), 57.2% (6 months), and 57.2% (final visit) of patients (Figure 2). The mean FRT significantly decreased from 369.1 *μ*m (±155.6 *μ*m) before treatment to 301.4 *μ*m (±146.3 *μ*m) (*P* < 0.01) after six weeks, to 281.3 *μ*m (±127.1 *μ*m) (*P* < 0.01) after six months and was 291.2 *μ*m (±168.7 *μ*m) (*P* < 0.01) at the final visit (Figure 3). The mean number of intravitreal bevacizumab injections was 3.71 (range 1–6).

## 4. Discussion

Anti-VEGF agents have produced better results than ever seen before in the treatment of exudative AMD. Both bevacizumab and ranibizumab, the most commonly used agents, are derived from the same murine monoclonal antibody against VEGF. Bevacizumab is a humanized monoclonal full-length antibody. Ranibizumab is the corresponding Fab fragment of the full-length anti-VEGF antibody. Both bevacizumab and ranibizumab bind VEGF at the same site and neutralize the biological activity of VEGF. The molecular similarity between the two agents strongly suggested that there would be a therapeutic similarity. Recently, many studies have reported comparisons of intravitreal ranibizumab versus bevacizumab for the treatment of AMD. Reviewing the literatures, there have been no significant differences between bevacizumab and ranibizumab, and both treatments seem to be effective for stabilizing VA loss [15–19]. However, there have been few studies documenting the effects of changing from one medication to another. Recently, a few studies have reported that there were no apparent differences in visual acuity after switching from bevacizumab to ranibizumab therapy [20, 21]. However, to the best of our knowledge, there has been no report documenting the results after switching from ranibizumab to bevacizumab therapy.

 In the present study, patients were treated initially with monthly intravitreal ranibizumab. During the course of treatment, we switched patients from ranibizumab to bevacizumab in the cases that needed to discontinue the intravitreal ranibizumab treatment because of its high cost. There was no statistically significant difference in the course of BCVA after switching to bevacizumab, while a stable or improved BCVA was achieved in 100% of subjects at six weeks, 57.2% at six months, and 57.2% at the final evaluation. With regard to the mean FRT, there was a statistically significant decrease after switching from ranibizumab until the final examination. The present study suggests that switching from ranibizumab to bevacizumab may be effective for maintaining BCVA and reducing the FRT, and for controlling the disease at least during the short-term followup period. 

 Despite the similarities between bevacizumab and ranibizumab, the treatment response differs between the drugs. Although the molecular size of ranibizumab and bevacizumab and their half life in vitreous fluid are different [22, 23], the etiology of the different responses is unknown. In this study, switching from ranibizumab to bevacizumab therapy did not appear to show any advantage or disadvantage in the course of BCVA, but bevacizumab appears to show an advantage over ranibizumab in the course of the mean FRT. Although there were isolated individuals who responded better to one drug or the other, in patients who are unable to continue the treatment with ranibizumab due to the high cost, switching from ranibizumab to bevacizumab may be a promising therapeutic option. 

 There were a few limitations to this study, including the fact that it was a retrospective study, that only a small number of patients were reviewed, and that the followup periods were relatively short. A prospective study with a large series of patients and controls may be necessary in order to determine whether it is safe and effective to change from one medication to another.

## Figures and Tables

**Table 1:** Demographics and characteristics of patients.

Pt	Age	Sex	Duration of IVR treatment (months)	Number of IVR	Prior PDT	Duration of IVB treatment (months)	Number of IVB
1	79	Female	6	3	(+)	13	3
2	79	Female	7	3	(−)	12	1
3	66	Female	11	5	(−)	11	3
4	62	Male	8	5	(+)	9	3
5	76	Female	10	6	(−)	10	6
6	65	Female	10	6	(−)	10	4
7	68	Male	8	6	(−)	11	6

IVR: intravitreal ranibizumab; PDT: photodynamic therapy; IVB: intravitreal bevacizumab.
